# 

**Published:** 1895-01

**Authors:** 


### CONTENTS.

COMMUNICATIONS.

The Present and Future Status of the Dental Practitioner .......... 1
Local Anæsthesia .......... 7
Random Thoughts on our Specialty .......... 8
Hygiene of the Mouth .......... 11
Fiftieth Anniversary of the Discovery of Anæsthesia by Horace Wells 16
Shedding the Temporary Teeth .......... 27
Some Experimental Tests with Sterilizing Agents .......... 34
Monthly Digest .......... 44

EDITORIAL.

Bibliographical .......... 51

NOTICES.

Tri-State Meeting .......... 52

